# Cost analysis of improving emergency care for aged care residents under a Hospital in the Nursing Home program in Australia

**DOI:** 10.1371/journal.pone.0199879

**Published:** 2018-07-03

**Authors:** Lijun Fan, Bill Lukin, Jingzhou Zhao, Jiandong Sun, Kaeleen Dingle, Rhonda Purtill, Sam Tapp, Xiang-Yu Hou

**Affiliations:** 1 Department of Biostatistics and Epidemiology, School of Public Health, Sun Yat-sen University, Guangzhou, China; 2 School of Public Health and Social Work, Queensland University of Technology, Brisbane, Queensland, Australia; 3 Department of Emergency Medicine, Royal Brisbane and Women’s Hospital, Brisbane, Queensland, Australia; 4 Bureau of Investment Promotion, Wuwei City, Gansu Province, P. R. China; 5 Queensland Health, Brisbane, Queensland, Australia; National Yang-Ming University, TAIWAN

## Abstract

**Background:**

This study aims to examine the costs associated with a Hospital in the Nursing Home (HiNH) program in Queensland Australia directed at patients from residential aged care facilities (RACFs) with emergency care needs.

**Methods:**

A cost analysis was undertaken comparing the costs under the HiNH program and the current practice, in parallel with a pre-post controlled study design. The study was conducted in two Queensland public hospitals: the Royal Brisbane and Women’s Hospital (intervention hospital) and the Logan Hospital (control hospital). Main outcome measures were the associated incremental costs or savings concerning the HiNH program provision and the acute hospital care utilisation over one year after intervention.

**Results:**

The initial deterministic analysis calculated the total induced mean costs associated with providing the HiNH program over one year as AU$488,116, and the total induced savings relating to acute hospital care service utilisation of AU$8,659,788. The total net costs to the health service providers were thus calculated at -AU$8,171,671 per annum. Results from the probabilistic sensitivity analysis (based on 10,000 simulations) showed the mean and median annual net costs associated with the HiNH program implementation were -AU$8,444,512 and–AU$8,202,676, and a standard deviation of 2,955,346. There was 95% certainty that the values of net costs would fall within the range from -AU$15,018,055 to -AU$3,358,820.

**Conclusions:**

The costs relating to implementing the HiNH program appear to be much less than the savings in terms of associated decreases in acute hospital service utilisation. The HiNH service model is likely to have the cost-saving potential while improving the emergency care provision for RACF residents.

## Introduction

The rising expenditures on hospital healthcare have been at the forefront of discussions among health professionals, economists and politicians [1–3]. Many countries, including the United States, Europe, and Australia, have reported a faster growth in health expenditure than that in the broader economy over recent years [1,4]. Yet it remains a demanding challenge to reduce people’s reliance on seeking for the expensive hospital care whenever they feel physically unwell. This applies especially among the very elderly people such as those living in the residential aged care facilities (RACFs), who represent one of the most vulnerable population disproportionately occupying the hospital resources [5]. They are usually borne with multiple chronic diseases prone to deteriorations and thus require frequent presentations to the emergency departments (EDs) and admissions to the acute hospitals [6].

Evidence suggests that opportunities exist to achieve healthcare savings by improving the emergency care services directed at the group of RACF patients [7–10]. A considerable proportion of hospital acute service utilisation by RACF residents is actually believed to be unnecessary or unsuitable [11–13]; and even worse, there is high chance of a range of traumatic hospital-acquired complications to arise during patient transfers to the acute hospital environment [14]. Hospital in the Nursing Home (HiNH) program was one of such policy initiatives in Queensland Australia, emphasizing on avoiding (or reducing the length of) the emergency hospital care among RACF residents. The HiNH scheme, funded by the state government, involves a team of two or three ED-based nurses (as the main program staff) coordinating with the RACF staff, general practitioners (GPs) and other health professionals to plan for the medical and nursing care for individual RACF patients. The program aims to avoid those unnecessary transfers to EDs and acute hospitals among RACF residents, by increasing RACF staff’s competency in delivering some relatively unsophisticated acute care within their own facility as an alternative to transfers to acute hospital settings. It also manages to discharge the RACF patients earlier back to their facilities, in a way of fast tracking the patients through the hospital system and facilitating panel communications on appropriate care planning for patients. The HiNH program works in every stage from RACFs to the EDs and acute inpatient wards to improve the care continuity for aged care residents.

Debate about the cost-saving potential of the HiNH program while improving the emergency care for RACF residents has been ongoing. Given that health care resources are very scarce and costly, it is imperative to understand whether the HiNH intervention would represent a worthwhile public health investment. However no study has yet been conducted to evaluate the HiNH program on the basis of costs. Our study thus compared the costs under current practice and the HiNH scheme, to identify if any savings exist that may exceed or offset the upfront costs associated with implementing a HiNH program.

## Materials and methods

### Study population and setting

A cost analysis was undertaken comparing the cost differences in providing emergency care for RACF residents under the existing practice and the HiNH scheme, to identify whether or not the HiNH program was preferred on economic grounds. Two groups of population were selected for the purpose of this costing study: 1) RACF patients coming from the catchment areas of Logan Hospital (LH, control group), who received routine health care where the majority of acute conditions resulted in transfers to EDs and hospital discharges occurred at the usual time; and 2) RACF patients from the catchment areas of Royal Brisbane and Women’s Hospital (RBWH, intervention group), who were cared in accordance with the HiNH scheme aimed at reducing emergency hospital attendances and minimizing the length of hospital stays. Both RBWH and LH were principal referral public hospitals located in major cities in Queensland, Australia, where RBWH had implemented the HiNH program since February 2006 and LH had no similar interventions. Ethics approval for this study was granted from the Human Research Ethics Committee at Queensland University of Technology (ethics clearance number: 1000000457).

### Data collection and analysis

This study took three fundamental steps before deriving the annual net costs associated with implementing the HiNH program, i.e., identification of resource use data involved, quantification of accrued and averted resource uses, and valuation of each of the resource items. The included costs and savings in our study were taken as incremental changes over the existing practice, as the HiNH intervention was implemented based on the current healthcare service system and focus of this study was on the cost changes owing to the intervention rather than the individual costs of the two alternatives. This costing analysis was conducted mainly from the perspective of healthcare providers.

#### A. Identification, quantification and valuation of resource use data

a) Identification: We identified the changes in net costs associated with one-year exposure to the HiNH intervention, which could be divided into two categories: 1) HiNH program costs: the induced costs due to incremental resource inputs for carrying out the HiNH program; and 2) hospital health service utilisation costs/savings: the induced costs/savings relating to the changes in acute hospital health service utilisation after implementing the intervention.

The HiNH program costs included both staffing costs and non-staff running costs. Staffing costs were those used for employing 2.6 full-time equivalent (FTE) nursing staff by the program. The time in kind offered by other health professionals such as staff specialist, geriatrician, pharmacist, social worker, etc., were also estimated and counted as staffing costs in this study. Non-staff running costs consisted of expenses of program staff travelling to and from RACFs (vehicle lease), administration and training, stationery and office suppliers, telephone communications, equipment (three computers, laptop, printer, fax, dect phone, three desk phones and two mobile phones), and office space.

The health service utilisation costs/savings were identified based on the increases/reductions associated with the HiNH program, in terms of ED presentation rate (i.e., number of ED presentations per 1,000 RACF beds per month), ED LOS, inpatient admission rate (i.e., number of inpatient admissions via ED per 1,000 RACF beds per month), inpatient LOS, and ambulance transport services between RACFs and acute hospitals. The costs of RACF beds remained unchanged before and after the HiNH intervention, and were thus not counted in the analysis. This was owing to the fact that while RACF residents were staying at EDs or inpatient wards, the funding for RACF beds provided by the Federal Government was still on-going despite the RACF beds being vacant.

b) Quantification and Valuation: Sources of quantification and valuation of the above identified resource use data were summarized in the last column of Table 1.

**Table 1:** Values of parameters used in cost analysis and their sources.

Parameter	Baseline estimate	Variation (s.e./range)	Assumed Distribution	Source
*HiNH program costs—staffing costs*, *in AU$ per annum*				
HiNH staff (including on-costs)	361,943	-	-	*
Time in kind from other health professionals	77,294	-	-	†
*HiNH program costs—non-staff costs*, *in AU$ per annum*				
Staff travel	6,835	-	-	‡
Administration and training	10,858	-	-	‡
Stationery and office suppliers	720	-	-	‡
Telephone communications	2,136	-	-	‡
Equipment (computer, laptop, printer, fax, phone)	19,100	-	-	§
Office space	9,230	-	-	(15) ^||^
*HiNH program costs—other parameters for sensitivity analysis*				
Useful life of equipment	-	0–10	Uniform	(22)
Annual discount rate	-	0–0.05	Uniform	(22)
**A: Subtotal (HiNH program costs)** = staffing costs+ non-staff costs				
*Hospital health service utilisation–parameters for calculating the amount of utilisation*				
a. ED presentation rate per month	63.19	6.58	Poisson	¶
b. Inpatient admission rate per month	49.77	7.11	Poisson	¶
c. ED LOS per presentation, hours	13.09	1.06	Gamma	¶
d. Inpatient LOS per admission, hours	80.22	23.28	Gamma	¶
e. Difference in ED presentation rate per month	-10.47	7.09	Normal	¶
f. Difference in inpatient admission rate per month	-23.48	7.35	Normal	¶
g. Difference in ED LOS per presentation, hours	-6.14	0.99	Normal	¶
h. Difference in inpatient LOS per admission, hours	-15.27	17.09	Normal	¶
i. Percentage of patients arriving by ambulance	0.5	±25%	Uniform	**
j. No. of RACF beds/1,000 RACF beds	2.485	-	-	‡
*Hospital health service utilisation–parameters for unit cost of utilisation*, *in AU$*				
k. Average ED presentation cost per hour	193	±25%	Triangular	(16, 17)
l. Average inpatient cost per hour	70	±25%	Triangular	(16)
m. Ambulance services cost per incidence	679	±25%	Triangular	(18)
**B: Subtotal (Costs for differences in hospital health service utilisation)** = [(e*c+a*g)*k+(f*d+b*h)*l+(e*i)*m]*j*12				
**C: Total (Net costs associated with the HiNH intervention)** = A+B				

* Based on data obtained from the hospital financial department.

^†^ Based on data obtained from panel consensus and Queensland Health wage rates.

^‡^ Based on data obtained from Hospital in the Nursing Home program record.

^§^ Estimated based on market prices.

^||^ Adjusted for inflation to 2011 AU dollars using the Australian government’s consumer price index.

^¶^ Based on data retrieved from EDIS & HBCIS and were assumed from the statistical modelling.

** A conservative estimation based on panel consensus.

We obtained cost data for the HiNH program from the hospital financial department and the program records, for the duration from March 2011 to February 2012. Equipment costs were estimated according to market prices. The initial deterministic analysis applied a relatively extreme lifespan assumption for the equipment, i.e., they were useful for one year and discarded after that. Costs for health professional resources provided in-kind and for office space were assumed based on other published sources (Queensland Health wage rates and previous study from Gray et al. (2009) [15]), and were indexed to 2011 prices. Valuation of the unit costs for ED stay, inpatient stay and ambulance transport were obtained from the published government reports [16–18]. Regarding the percentage of ambulance patients, this study used a conservative estimation of 50% for the initial calculation based on panel consensus.

Sources of data regarding hospital health service utilisation were based on the patient records retrieved from electronic hospital databases including Emergency Department Information System (EDIS) and Hospital Based Corporate Information System (HBCIS) and the predictions from statistical modelling. Further details on data collection for this part could be found in another accompanying paper from Fan et al. (2016) [19]. We analysed the differences in acute health resource services utilisation associated with the HiNH program surrounding a pre-post controlled study design, where evaluation was conducted over three-month pre-test period (June to August 2005) and three-month post-test period (June to August 2011) for one intervention hospital (RBWH) and one control hospital (LH). Generalized linear models (GLMs) were applied to analyse the changes in acute care utilisation associated with the HiNH implementation. The proposed models were written as: Y = g [μ(x_1_, x_2_, x_3_)] = β_0_ + β_1_ x_1_ + β_2_ x_2_ + β_3_ x_3_ + β_4_ x_4_ + ε_i_, where x1 represented Hospital (x_1_ = 0, Hospital = LH; x_1_ = 1, Hospital = RBWH), x_2_ represented Year (x_2_ = 0, Year = 2005; x_2_ = 1, Year = 2011), x_3_ represented Intervention (x_3_ = 0, no intervention; x_3_ = 1, with intervention), x_4_ represented a series of confounders (such as age, gender, etc.), and ε_i_ was a vector of unknown residuals. The appropriate models were selected based on how the distributional assumption fit the data and which approach yielded the best residual. Analysis on ED presentation rate and inpatient admission rate were achieved with Poisson log-linear models. To model ED LOS and inpatient LOS outcomes, we chose GLMs using gamma distribution and log link function as recommended [20,21], while controlling for confounders including patient age, gender, triage scale, primary diagnosis, day and time of hospital attendances. The gamma distribution is similar to the log-normal distribution in shape, and is robust to characterize the non-normal distributions typical of the LOS data (non-zero and highly positively skewed). The log link function prevented the negative prediction of the outcome. Diagnostic plots and statistics of the residuals for LOS outcomes were also generated and checked to ensure that the residuals from fitted models were close to normal distributions with no serious outliers.

All regression coefficients from the fitted models were then exponentiated to obtain the ratio in the given outcome variable associated with a specific change in each independent variable. The exp (β3) was used to quantify the intervention impact on the outcome variables. The adjusted means of ED presentation rate (per month), inpatient admission rate (per month), ED LOS (hour), and inpatient LOS (hour) for a given group were also predicted based on the fitted models and controlled for all other variables included in the models. Although LOS data were highly skewed, we still presented the means for them instead of the medians, because provision of the medians was considered unhelpful for service planners interested in the cost estimates.

#### B. Cost analysis: Deterministic analysis and sensitivity analysis

The parameters related to cost differences between the existing practice and the HiNH scheme were available through the process of identification, quantification and valuation of all related resource use data, and all parameters were listed in Table 1.

Then we used the following equations to summarize the annual net costs associated with the HiNH implementation. Any costs with negative values meant “savings” and any costs with positive values meant “losses”.

$$\mathrm{Net}\;\mathrm{costs}=A_{\left(\mathrm{HiNH}\;\mathrm{program}\;\mathrm{costs}\right)}+B_{\left(\mathrm{Costs}\;\mathrm{for}\;\mathrm{differences}\;\mathrm{in}\;\mathrm{hospital}\;\mathrm{health}\;\mathrm{service}\;\mathrm{utilisation}\right)}$$

where:

A=A1(HiNHprogram:staffingcosts)+A2(HiNHprogram:non-staffcosts)B=B1(CostsfordifferencesinEDcareutilisation)+B2(Costsfordifferencesininpatientcareutilisation)+B3(Costsfordifferencesinambulanceserviceutilisation)=[(e*c+a*g)*k+(f*d+b*h)*l+(e*i)*m]*j*12

Therefore, the net costs arising from one-year implementation of HiNH program as compared with the current practice were obtained, where a negative value of net costs represented “cost-saving” and a positive value represented “not cost-saving”.

The robustness of cost findings was explored with a further sensitivity analysis. Sensitivity analysis was considered as a main way of dealing with uncertainty in input parameters and allowed for examining the possible generalizability of results to other similar settings. In this study, a Monte Carlo simulation approach (based on 10,000 times of simulations) was performed to assess how the modelling results of net costs changed when input parameters were varied across a plausible range. The assumptions of variations and probability distributions around each input parameter were presented in Table 1.

In the sensitivity analysis, the equipment was assumed to have a useful life varying from 0 to 10 years and an annual discount rate from 0 to 0.05 [22]. Measurements of the acute hospital care utilisation (ED presentation and inpatient admission related data) were varied according to the indicated range of values derived from the modelling. Other parameters with uncertainties were adjusted up and down by 25% over their baseline estimates [23]. A method of moments approach was used to fit the gamma distribution given the mean and standard error that were reported.

Analyses were performed using SPSS v. 21 (IBM Corp., Armonk, NY) and Microsoft Excel with an add-in program of Oracle Crystal Ball. Two tailed tests were used and p values of ≤ .05 were considered significant. All prices were measured in Australian Dollars (AU$).

## Results

### Results on models quantifying changes in hospital emergency care utilisation

Table 2 presents comparisons of sociodemographic and clinical characteristics of the patients included for analysis. The majority of patients were the elderly aged above 65 years old (otherwise they would presumably be injured or chronically unwell which thus still required aged care), were female and with relatively low triage acuity (ATS 3–5), and they most arrived at EDs during weekdays and working hours. No differences were found between groups in terms of patient age, gender, ATS, day and time of hospital visits; while some differences were identified regarding patients’ primary diagnosis leading to ED presentations. For example, among all RACF patients who had hospital ED presentations, those presenting to RBWH after intervention had lower percentage of respiratory, circulatory, and digestive related diagnoses, but higher percentage of injury and poisoning, and genitourinary related diagnoses, compared with patients presenting to RBWH before intervention or to the control hospital during the post-test period.

**Table 2:** Comparison of patients' demographic and clinical characteristics.

Indicators	ED presentation Cohort					Hospital admission via the ED Cohort				
	Number of ED presentation in this category			p-value		Number of hospital admission in this category			p-value	
	RBWH Pre-test (n = 449)	RBWH Post-test (n = 393)	LH Post-test (n = 265)	RBWH-Pre vs. RBWH-Post	RBWH-Post vs. LH-Post	RBWH Pre-test (n = 256)	RBWH Post-test (n = 196)	LH Post-test (n = 168)	RBWH-Pre vs. RBWH-Post	RBWH-Post vs. LH-Post
Age Group, n (%)				0.997	0.424				0.913	0.465
<65^†^	29 (6.5)	26 (6.6)	24 (9.1)			9 (3.5)	10 (5.1)	15 (8.9)		
65–74	58 (12.9)	49 (12.5)	43 (16.2)			32 (12.5)	21 (10.7)	22 (13.1)		
75–84	137 (30.5)	123 (31.3)	81 (30.6)			81 (31.6)	63 (32.1)	50 (29.8)		
85–94	193 (43.0)	169 (43.0)	101 (38.1)			113 (44.1)	86 (43.9)	72 (42.9)		
≥95	32 (7.1)	26 (6.6)	16 (6.0)			21 (8.2)	16 (8.2)	9 (5.4)		
Gender, n (%)				0.887	0.567				0.239	0.329
Male	168 (37.4)	145 (36.9)	104 (39.2)			88 (34.4)	78 (39.8)	58 (34.5)		
Female	281 (62.6)	248 (63.1)	161 (60.8)			168 (65.6)	118 (60.2)	110 (65.5)		
Australasian Triage Scale^‡^, n (%)				0.480	0.921				0.225	0.906
ATS 1&2 (seen immediately)	80 (17.8)	78 (19.8)	54 (20.4)			57 (22.3)	54 (27.6)	45 (26.8)		
ATS 3–5 (can wait)	369 (82.2)	315 (80.2)	211 (79.6)			199 (77.7)	142 (72.4)	123 (73.2)		
Attendance Day, n (%)				1.000	0.320				0.467	1.000
Weekday	339 (75.5)	296 (75.3)	190 (71.7)			184 (71.9)	134 (68.4)	114 (67.9)		
Weekend	110 (24.5)	97 (24.7)	75 (28.3)			72 (28.1)	62 (31.6)	54 (32.1)		
Attendance Time, n (%)				0.105	0.151				0.687	0.504
Working hours	284 (63.3)	227 (57.8)	138 (52.1)			86 (33.6)	62 (31.6)	59 (35.1)		
After hours	165 (36.7)	166 (42.2)	127 (47.9)			170 (66.4)	134 (68.4)	109 (64.9)		
Primary Diagnosis, n (%)				0.003**	0.004**				0.007**	0.017*
Injury & Poisoning	104 (23.2)	108 (27.5)	58 (21.9)			59 (23.0)	40 (20.4)	31 (18.5)		
Respiratory	51 (11.4)	39 (9.9)	47 (17.7)			36 (14.1)	27 (13.8)	37 (22.0)		
Circulatory	42 (9.4)	31 (7.9)	27 (10.2)			28 (10.9)	20 (10.2)	21 (12.5)		
Digestive	40 (8.9)	14 (3.6)	23 (8.7)			27 (10.5)	6 (3.1)	17 (10.1)		
Genitourinary	22 (4.9)	29 (7.4)	18 (6.8)			12 (4.7)	21 (10.7)	10 (6.0)		
Musculoskeletal & Skin	38 (8.5)	18 (4.6)	10 (3.8)			20 (7.8)	9 (4.6)	8 (4.8)		
Mental & Neurological	23 (5.1)	17 (4.3)	10 (3.8)			6 (2.3)	9 (4.6)	6 (3.6)		
Other	129 (28.7)	137 (34.9)	72 (27.2)			68 (26.6)	64 (32.7)	38 (22.6)		

** p<0.01

* p<0.05.

† Please note here that we did not exclude for analysis those patients aged less than 65 years old who had lived at RACFs and had presentations to hospital EDs; because although these patients were at relatively younger ages (mean ± SD: 56.9 ± 10.8 years; range from 21 to 64 years), they were in aged care (presumably owing to their being injured or otherwise chronically unwell)

‡ ATS 1&2: Patients who must be seen immediately; ATS 3–5: Patients who can wait and will be seen in order of arrival.

Results on the models to analyse the association of outcome variables (i.e., ED presentation rate, inpatient admission rate, ED LOS, and inpatient LOS) with independent variables are presented in Table 3. The exponentiated coefficients along with their 95% CIs and p-values for the main independent variables were displayed. According to the models, while holding all other variables constant, implementing the HiNH intervention, as compared with the existing practice, was associated with lower chances of ED presentations (rate ratio: 0.83 (95% CI, 0.67 to 1.05), p = 0.117) and hospital admissions (rate ratio: 0.53 (95% CI, 0.39 to 0.72), p = <0.0001), and were likely to show decreases in ED LOS (ratio: 0.53 (95% CI, 0.46 to 0.62), p = <0.0001) and inpatient LOS (ratio: 0.81 (95% CI, 0.53 to 1.23), p = 0.323). Results on the predicted means of response variables for the given groups after adjusting for all other variables included in the model are also summarized in Table 3.

**Table 3:** The fitted generalized linear models on response variables.

Response variable	Model	Exp(β), 95% CI^‡^	p-value	Adjusted mean^§^ (95% CI)
ED presentation rate*	Hospital	0.94 (0.80, 1.11)	0.455	-
	Year	0.90 (0.75, 1.08)	0.246	-
	Intervention	0.83 (0.67, 1.05)	0.117	
	RBWH-2011			52.72 (47.75, 58.19)
	RBWH-2005			70.37 (64.15, 77.18)
	LH-2011			67.28 (59.64, 75.88)
	LH-2005			74.92 (65.38, 85.85)
Inpatient admission rate*	Hospital	1.17 (0.92, 1.48)	0.199	-
	Year	1.24 (0.96, 1.60)	0.093	-
	Intervention	0.53 (0.39, 0.72)	<0.0001	
	RBWH-2011			26.29 (22.86, 30.24)
	RBWH-2005			40.12 (35.49, 45.35)
	LH-2011			42.65 (36.66, 49.61)
	LH-2005			34.38 (28.12, 42.04)
ED LOS^†^, hours	Hospital	1.37 (1.22, 1.54)	<0.0001	-
	Year	2.28 (2.02, 2.59)	<0.0001	-
	Intervention	0.53 (0.46, 0.62)	<0.0001	
	RBWH-2011			6.95 (6.26. 7.72)
	RBWH-2005			5.73 (5.20, 6.32)
	LH-2011			9.56 (8.54, 10.70)
	LH-2005			4.19 (3.70, 4.74)
Inpatient LOS^†^, hours	Hospital	1.10 (0.79, 1.52)	0.581	-
	Year	0.85 (0.61, 1.19)	0.335	-
	Intervention	0.81 (0.53, 1.23)	0.323	
	RBWH-2011			64.95 (42.00, 100.45)
	RBWH-2005			94.53 (61.48, 145.36)
	LH-2011			73.19 (47.53, 112.69)
	LH-2005			86.24 (54.97, 135.30)

* ED presentation rate: Number of ED presentations per 1,000 RACF beds per month.

Inpatient admission rate: Number of inpatient admissions via ED per 1,000 RACF beds per month.

ED presentation rate and inpatient admission rate were analysed using Poisson log-linear models.

^†^ ED LOS and inpatient LOS were analysed using generalized linear models (GLMs) with gamma distribution and log link, while adjusting for confounders including patients’ age, gender, Australasian Triage Scale (ATS), primary diagnosis, day (weekday/weekend) and time (working hours/after hours) of patients’ hospital attendances.

^‡^ CI: Confidence interval.

^§^ Adjusted means were the predicted means adjusted for all other variables in the model.

**Fig 1:**
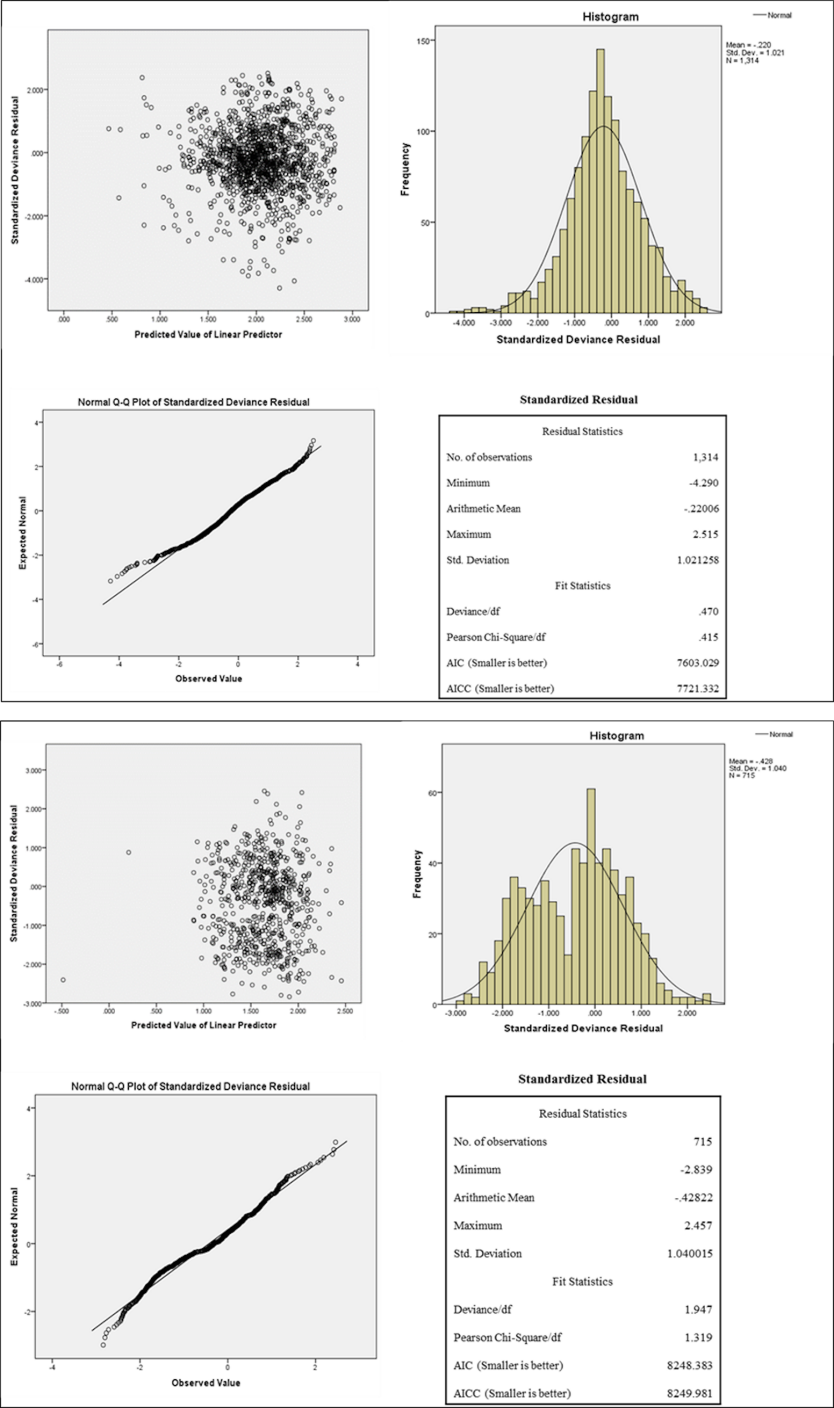
Residual diagnostics: Residuals for ED LOS (upper figure) and inpatient LOS (lower figure).

For the purpose of checking the fitness of selected models on the outcome LOS, diagnostic plots (including a scatter plot of the standardized residuals against the predicted values, a histogram with a normal density curve, and a Q-Q plot) and statistics of the residuals obtained from the models are shown in Fig 1. The upper figure described the residuals for ED LOS and the lower figure was for the residuals of inpatient LOS. Results showed that for both models, the points in the scatter plots fluctuated randomly around zero with no obvious patterns, the histograms were roughly symmetrical and bell-shaped, and the Q-Q plots conformed a roughly straight line. Residual statistics showed the deviance/df and Person Chi-Square/df approximated to one. The diagnoses indicated that the residuals produced from the models on ED and inpatient LOS were relatively good.

Statistical inferences regarding effectiveness of the HiNH program on the response variables were made based on the fitted models, and the results are presented in Table 4. If the HiNH intervention was not introduced to RBWH in 2011, the estimated values of the outcome variables were estimated to be 63.19 (95% CI, 51.53 to 77.48) for the number of ED presentations per 1,000 RACF beds per month (ED presentation rate), 49.77 (95% CI, 37.62 to 65.84) for the number of inpatient admissions per 1,000 RACF beds per month (inpatient admission rate), 13.09 (95% CI, 11.16 to 15.36) hours for the average ED LOS, and 80.22 (95% CI, 45.42 to 141.69) hours for the average inpatient LOS. Implementing the HiNH intervention was likely to be associated with approximately 10 ED presentations (Mean (95% CI): -10.47 (-32.58 to 11.64); p = 0.117) and 23 hospital admissions (Mean (95% CI): -23.48 (-46.38 to -0.57); p<0.0001) avoided per 1,000 RACF beds per month; and the ED LOS and inpatient LOS per episode would be likely to decrease by 6.14 hours (95% CI, -9.22 to -3.06; p<0.0001) and 15.27 hours (95% CI, -68.53 to 38.00; p = 0.323), respectively. These estimations were used for the further costing analysis as a measurement of the differences in the amount of acute hospital care service utilisation due to the HiNH implementation.

**Table 4:** Differences in response variables with and without the HiNH intervention, estimated for RBWH for the year 2011–12.

Response variable	Without-intervention estimate, Mean (95% CI)	With-intervention estimate, Mean (95% CI)	Mean difference associated with the intervention, Mean (95% CI)	p-value
ED presentation rate*	63.19 (51.53, 77.48)	52.72 (47.75, 58.19)	-10.47 (-32.58, 11.64)	0.117
Inpatient admission rate*	49.77 (37.62, 65.84)	26.29 (22.86, 30.24)	-23.48 (-46.38, -0.57)	<0.0001
ED LOS, hours	13.09 (11.16, 15.36)	6.95 (6.26. 7.72)	-6.14 (-9.22, -3.06)	<0.0001
Inpatient LOS, hours	80.22 (45.42, 141.69)	64.95 (42.00, 100.45)	-15.27 (-68.53, 38.00)	0.323

* ED presentation rate: Number of ED presentations per 1,000 RACF beds per month.

Inpatient admission rate: Number of inpatient admissions via ED per 1,000 RACF beds per month.

### Results on identification, quantification and valuation of resource use data

All identified changes in resource uses while implementing the HiNH program versus the existing practice are summarized in Table 1. A list of all parameters relating to resource uses necessary for the subsequent cost analysis was presented, and the results on quantification and valuation of each resource item were displayed and derived from the sources indicated in Table 1. For the purpose of sensitivity analysis, variations and assumed probability distributions were assigned to all parameters, in accordance with either data characteristics or model predictions (Table 1).

### Results on cost analysis

#### Deterministic analysis

Results of the initial deterministic analysis are summarized in Table 5. This analysis calculated the total induced mean costs associated with implementing the HiNH program over one year as $488,116, where the salary paid for the program staff took up the largest proportion ($361,943, 74.15%). Results showed there were associated savings in terms of acute care service utilisation, reaching a mean saving of $8,659,788 in total per annum. Among them, the largest saving might be owing to the reduction in patient stay at inpatient wards (3,936,270+1,588,221 = $5,524,491, 63.79%), followed by the decrease in patient stay at EDs (790,746+2,238,554 = $3,029,300, 34.98%) and then the avoided ambulance transport between RACFs and acute hospitals ($105,997, 1.22%). To sum up, over one-year implementation of the HiNH program, the total net costs to the health service providers were estimated to be -$8,171,671.

**Table 5:** Summary of induced net costs per annum associated with the HiNH program.

Resource item	Cost, AU$	
*A1*: *HiNH program costs—staffing costs*		*439*,*237*
HiNH staff (including on-costs)	361,943	
Time in kind from other health professionals	77,294	
*A2*: *HiNH program costs—non-staff costs*		*48*,*879*
Staff travel	6,835	
Administration and training	10,858	
Stationery and office suppliers	720	
Telephone communications	2,136	
Equipment (computer, laptop, printer, fax, phone)	19,100	
Office space	9,230	
**A: Subtotal (HiNH program costs)** = A1+A2		**488,116**
*B1*: *Cost for differences in ED care utilisation*		*-3*,*029*,*300*
Cost for avoided ED presentation bed-hours = (e*c*k)*j*12	-790,746	
Costs for shortened ED presentation bed-hours = (a*g*k)*j*12	-2,238,554	
*B2*: *Cost for differences in inpatient care utilisation*		*-5*,*524*,*491*
Cost for avoided inpatient admission (via ED) bed-hours = (f*d*l)*j*12	-3,936,270	
Costs for shortened inpatient admission (via ED) bed-hours = (b*h*l)*j*12	-1,588,221	
*B3*: *Cost for differences in ambulance service utilisation =* (e*i*m)*j*12		*-105*,*997*
**B: Subtotal (Costs for differences in hospital health service utilisation)** = B1+B2+B3		**-8,659,788**
**C: Total (Net costs associated with the HiNH intervention)** = A+B		**-8,171,671**

#### Probabilistic sensitivity analysis

**Fig 2:**
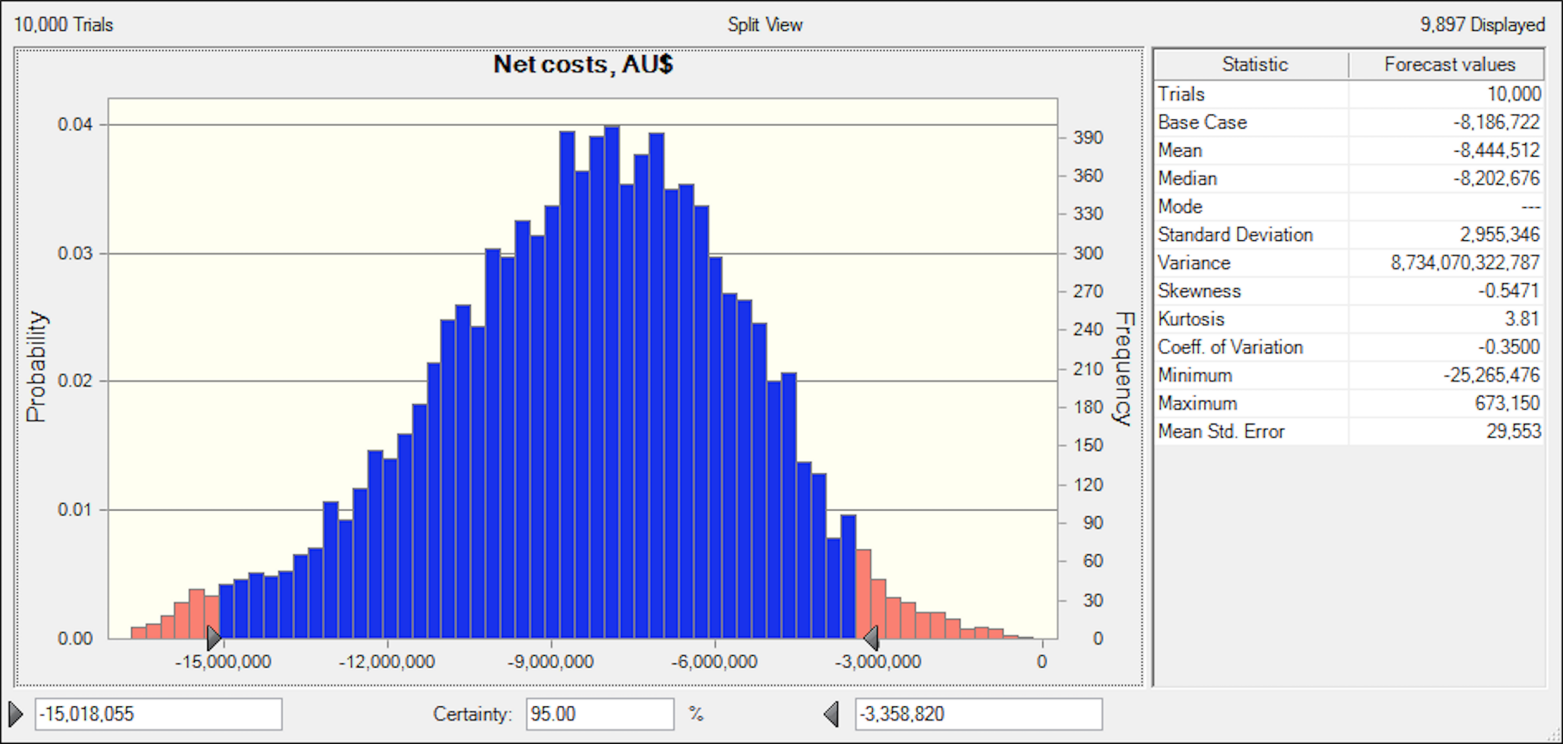
Monte Carlo simulation results for the net costs per annum associated with the HiNH program.

Monte Carlo simulation approach was conducted to explore the impact of uncertainty in input parameters on the modelling outcome of net costs. The results from 10,000 simulations were presented in Fig 2. Based on the 10,000 trials, the mean and median annual net costs associated with the HiNH program implementation were -$8,444,512 and -&8,202,676, and a standard deviation of 2,955,346. The minimum and maximum value for net costs from these simulations were -$25,265,476 and $673,150. There was 95% certainty that the values of net costs would fall within the range from -$15,018,055 to -$3,358,820.

**Fig 3:**
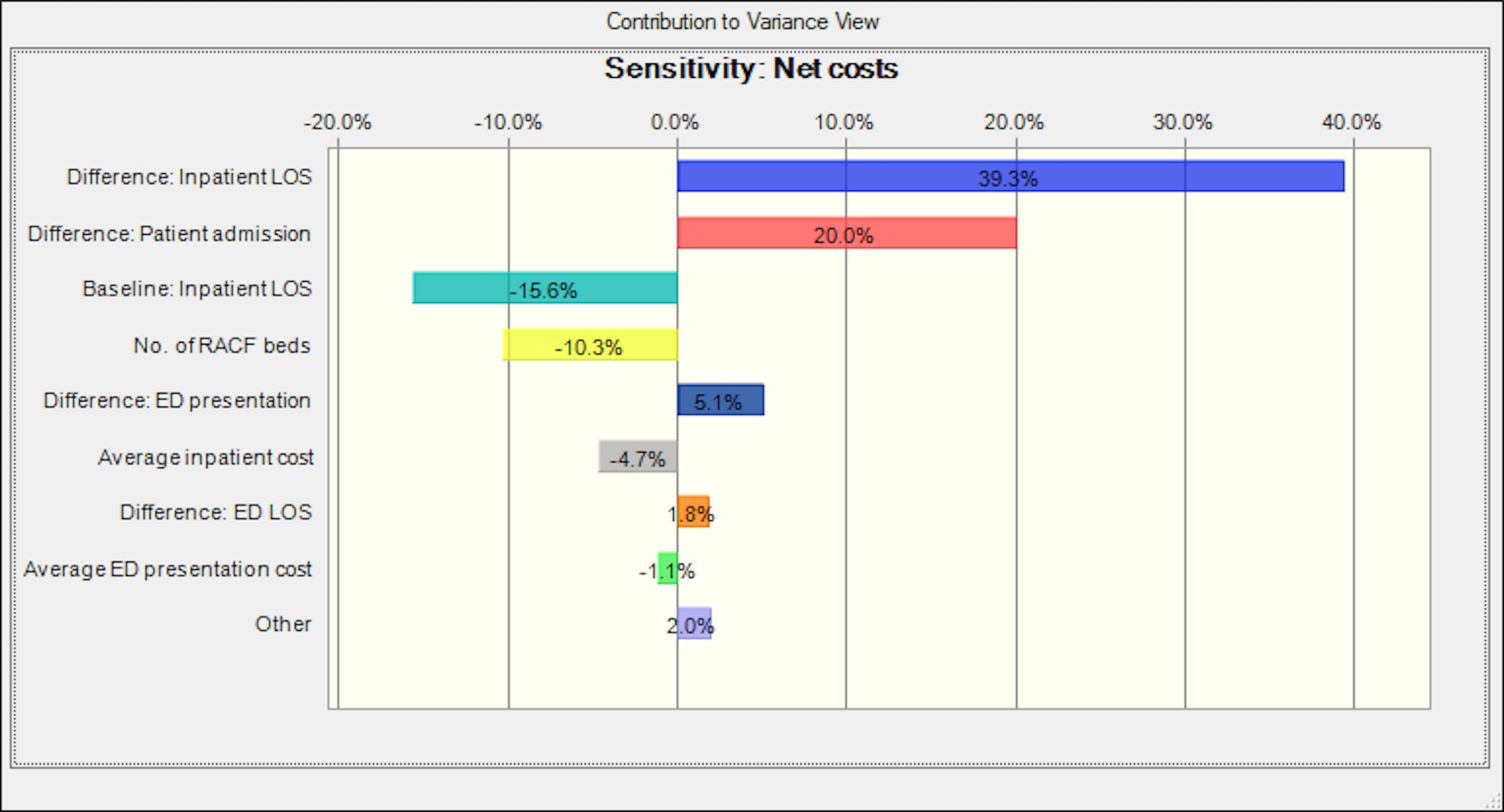
Sensitivity chart showing main parameters that contributed to the variance in values of net costs.

A sensitivity chart showing the effects of input parameters on the modelling results of net costs was also presented (Fig 3). The variations in the parameter of “difference in inpatient LOS due to the HiNH implementation” and the parameter of “difference in inpatient admission rate due to the HiNH implementation” accounted for 39.3% and 20.0% of the variance in the simulated net costs, respectively; and an increase in these two parameters would lead to an increase in net costs. Another parameter (“baseline estimate of average inpatient LOS at RBWH in 2011 if no intervention”) was ranked as the third major contributor to the variance in results, accounting for 16.6% of the variance and had a negative correlation with the results. Other input parameters contributed very little to the variance of results, each of which accounted for around or less than 10% of variance in outcome values.

## Discussion

Although policy interest has been placed for years in lowering the reliance on acute hospital service utilisation from RACF residents, little research has reported the associated costs. To our knowledge, Hospital in the Nursing Home (HiNH) program in Queensland Australia, as such a policy initiative, has not yet received any evaluation on the basis of costs. The present study thus provides the first evidence on the costing aspects of the HiNH model of service delivery that aims to lower the incidence rates of potentially avoidable RACF transfers to EDs and acute hospitals and to reduce the length of ED and inpatient stay by RACF patients when they do attend the hospitals.

Results of this study reveal that implementing the HiNH program was likely to be associated with substantial cost savings to the health care provider. Although the average costs for providing the HiNH services reached $488,116 per annum, this cost appeared to be more than offset by the $8,659,788 annual savings in the acute hospital care utilisation by RACF patients. Considerably due to this difference in acute hospital care-related savings, the net costs over one-year period of intervention were estimated to be $8,171,671 lower for interventions than for controls. The further sensitivity analysis has, under all assumptions used and based on 10,000 trials, concluded a consistent result that the incremental net cost associated with the intervention has always been negative (meaning the intervention is cost-saving) with very few exceptions. This work adds an important empirical dimension to the current debate regarding economic feasibility of the program. Results from the rigorous evaluations may have an immediate translational impact on guiding the health resource allocations to an ageing population. Our findings suggest that application of the HiNH service model may represent a potentially cost-saving method of coping with the overuse of emergency hospital resources by RACF residents, with fewer additional resources required for carrying out the program than would otherwise be needed to increase the accessibility to acute hospital care for the RACF patients.

Our findings support that of others where coordinated approaches in hospital avoidance or substitution programs have contained the healthcare costs by decreasing patients’ demand for ED or acute inpatient beds [2, 12, 24–27]. These approaches encompass measures to improve the availability of primary or community care so that patients otherwise require hospital transfers could be alternatively cared at their residencies or in the community, and measures of fast tracking and early discharge planning for patients visiting hospitals so that they could depart from the ED or hospital at an earliest time. A large literature has been establishing the possibility of such measures toward reductions in hospital attendances and decrease in length of hospital stays [28,29], both of which would bring about savings in acute hospital service utilisation and thus related costs. Associated savings in hospital care service utilisation are considered as a fundamental driver of the cost-saving potential of such programs; and by this means, hospital resources can be more appropriately and efficiently used by other people in needs.

Whilst some studies have documented a potential for cost savings of the programs[2, 12, 24–27], others have not [3, 9, 30]. These studies reported the programs as either cost-neutral or more costing. The major reasons include that, although patients have a shorter length of stay at hospitals, they require alternate care at homes or communities and the overall duration of an episode of care is actually increased. Patients are usually discharged early from hospitals when their hospital care is least expensive, while the nursing costs outside the hospital environment still have to be incurred. It seems unlikely under such a situation to reduce the overall costs, because costs are simply shifted from one location to another. The HiNH program under investigation of this study, however, is not such a case. Under the HiNH scheme, RACF beds are funded by the Federal Government, while ED and inpatient beds are funded by the State Government. The HiNH program is also funded by the State Government, which may not be surprising because RACF residents under the HiNH intervention are actually receiving hospital-type care in the RACFs which otherwise requires ED and inpatient bed services. The difference we discuss here is that, the costs for RACF beds funded by the Federal Government are always on-going, even when the RACF patients are at the hospitals and the RACF beds are vacant. That means the costs of RACF beds would remain unchanged with and without the HiNH intervention; however, while patients are in EDs or hospitals, both the Federal Government and the State Government are funding a bed for the patient—the vacant RACF bed and the occupied hospital bed, and this is where significant cost savings could be made. It may be on the basis of above evidence that the HiNH program is considered to have different results compared with those from similar programs. We speculate the HiNH program would appear to have cost advantages, provided that it shows effectiveness in reducing acute health service utilisation and monetary values of the reductions exceed the direct costs of the program itself. Additional research which has found a higher cost on average for hospitalisations of RACF residents than for hospitalisations of community dwelling, would further add to the strength of our assumption of the program’s saving potential. Overall, it is important to understand that HiNH is a heterogeneous term that cannot be defined consistently across studies, and evaluation of HiNH should always focus on the fundamental components of the program strategies in order to reach an accurate conclusion.

### Limitations

Several limitations of this study are acknowledged. Firstly, within the study design to quantify impact of the HiNH intervention on the acute hospital attendance and stay among RACF patients, it was impractical for this study to randomise patients to the program groups given its implementation in the natural setting and was unable to analyse the role of different RACFs. Our current study could not conclude any causality, but could indicate the potential associations. Besides, inevitably there might be bias between the baseline patient groups, thus we attempted to adjust for the factors relating to patient demographics and clinical characteristics while analysing the outcome of length of stay. Secondly, this evaluation was conducted in two hospitals in Queensland Australia, and our findings may not be representative of those from other settings, considering the variety in the type, size, scope, and organisational issues of institutions. However, the consistent results obtained from sensitivity analysis have increased our confidence in generalizing findings of this study to other similar contexts. A wider roll-out of the intervention across multiple hospitals or regional areas might show different scenarios regarding the cost-saving magnitude, which thus warranted further multi-centre and large-scale studies to reinforce the generalisability of the HiNH model. Finally, this study examined a relatively short time span, thus it is still uncertain how the results would change over the time. Yet the HiNH intervention effect is supposed to be immediate given its nature, which may suggest that the short-term effect is likely to be representative in a longer run. The intervention itself might encounter a beginning short period with instability as it needed to recruit personnel not yet familiar to the program operation and to prepare for the infrastructure construction, etc., however, we assumed that it should come to a stable and sustainable status after that period. In this study, the year included for this cost analysis was approximately five years after the HiNH was first introduced, thus we speculate the effect in this year should represent a nearly stable status of the intervention.

## Conclusions

Our study indicates that the costs of providing the HiNH program appear to be significantly less than the savings in terms of associated decreases in acute hospital service utilisation. From the perspective of healthcare providers, the HiNH model is likely to have a cost-saving potential while simultaneously avoiding or reducing the phase of acute hospital care required by RACF residents. This study suggests that the HiNH service may act as a viable and worthwhile complement to hospital care in managing the demands for emergency department presentations and acute hospital admissions from RACFs. A wider-spread adoption of the program to other similar contexts may be supported on the basis of costs, yet requires further research to explore its actual expanded feasibility.

## References

[1] Robinson JC, Smith MD. Cost-reducing innovation in health care. Health Aff. 2008;27(5):1353–6. 10.1377/hlthaff.27.5.1353 18780924

[2] Coast J, Richards SH, Peters TJ, Gunnell DJ, Darlow M-A, Pounsford J. Hospital at home or acute hospital care? A cost minimisation analysis. BMJ. 1998;316(7147):1802–6. 9624074 10.1136/bmj.316.7147.1802 PMC28581

[3] Shepperd S, Harwood D, Gray A, Vessey M, Morgan P. Randomised controlled trial comparing hospital at home care with inpatient hospital care. II: cost minimisation analysis. BMJ. 1998;316(7147):1791–6. 9624069 10.1136/bmj.316.7147.1791 PMC28579

[4] Australian Institute of Health and Welfare. Australian's Health 2014. Canberra: AIHW; 2014.

[5] Jones JS, Dwyer PR, White LJ, Firman R. Patient transfer from nursing home to emergency department: outcomes and policy implications. Acad Emerg Med. 1997;4(9):908–15. 9305434 10.1111/j.1553-2712.1997.tb03818.x

[6] Crilly J, Chaboyer W, Wallis M, Thalib L, Polit D. An outcomes evaluation of an Australian Hospital in the Nursing Home admission avoidance programme. J Clin Nurs. 2011;20(7‐8):1178–87. doi: 10.1111/j.1365-2702.2010.03371.x 20846245 10.1111/j.1365-2702.2010.03371.x

[7] Rajacich DL, Cameron S. Preventing admissions of seniors into the emergency department. J Gerontol Nurs. 1995;21(10):36 7594249 10.3928/0098-9134-19951001-08

[8] Grabowski DC, O’Malley AJ, Barhydt NR. The costs and potential savings associated with nursing home hospitalizations. Health Aff. 2007;26(6):1753–61. 10.1377/hlthaff.26.6.1753 17978395

[9] Hensher M, Fulop N, Hood S, Ujah S. Does hospital-at-home make economic sense? Early discharge versus standard care for orthopaedic patients. J R Soc Med. 1996;89(10):548 8976887 10.1177/014107689608901003 PMC1295953

[10] Arendts G, Quine S, Howard K. Decision to transfer to an emergency department from residential aged care: A systematic review of qualitative research. Geriatr Gerontol Int. 2013;13(4):825–33. doi: 10.1111/ggi.12053 23506079 10.1111/ggi.12053

[11] Codde J, Arendts G, Frankel J, Ivey M, Reibel T, Bowen S, et al Transfers from residential aged care facilities to the emergency department are reduced through improved primary care services: An intervention study. Australas J Ageing. 2010;29(4):150–4. doi: 10.1111/j.1741-6612.2010.00418.x 21143359 10.1111/j.1741-6612.2010.00418.x

[12] Jones J, Wilson A, Parker H, Wynn A, Jagger C, Spiers N, et al Economic evaluation of hospital at home versus hospital care: cost minimisation analysis of data from randomised controlled trial. BMJ. 1999;319(7224):1547–50. 10591720 10.1136/bmj.319.7224.1547 PMC28300

[13] Arendts G, Reibel T, Codde J, Frankel J. Can transfers from residential aged care facilities to the emergency department be avoided through improved primary care services? Data from qualitative interviews. Australas J Ageing. 2010;29(2):61–5. doi: 10.1111/j.1741-6612.2009.00415.x 20553535 10.1111/j.1741-6612.2009.00415.x

[14] Bergman H, Carfield AM. Appropriateness of patient transfer from a nursing home to an acute-care hospital: a study of emergency room visits and hospital admissions. J Am Geriatr Soc. 1991;39(12):1164–8. 1960359 10.1111/j.1532-5415.1991.tb03568.x

[15] Gray LC, Wright OR, Cutler AJ, Scuffham PA, Wootton R. Geriatric ward rounds by video conference: a solution for rural hospitals. Med J Aust. 2009;191(11):605. 20028277 10.5694/j.1326-5377.2009.tb03345.x

[16] Independent Hospital Pricing Authority. National Hospital Cost Data Collection Australian Public Hospitals Cost Report 2011–2012, Round 16: Canberra (AUST): IHPA; 2012.

[17] Australian Institute of Health and Welfare. Australian Hospital Statistics 2011–12: Emergency Department Care. Canberra: AIHW; 2012.

[18] Queensland Government. Department of Community Safety Annual Report 2011–12: Queensland (AUST): Department of Community Safety, Queesland Government; 2012.

[19] Fan L, Hou X-Y, Zhao J, Sun J, Dingle K, Purtill R, et al Hospital in the Nursing Home program reduces emergency department presentations and hospital admissions from residential aged care facilities in Queensland, Australia: a quasi-experimental study. BMC Health Serv Res. 2016;16(1):1. 26857447 10.1186/s12913-016-1275-z PMC4746777

[20] Faddy M, Graves N, Pettitt A. Modeling length of stay in hospital and other right skewed data: comparison of phase-type, gamma and log-normal distributions. Value Health. 2009;12(2):309–14. doi: 10.1111/j.1524-4733.2008.00421.x 20667062 10.1111/j.1524-4733.2008.00421.x

[21] Manning WG, Basu A, Mullahy J. Generalized modeling approaches to risk adjustment of skewed outcomes data. J Health Econ. 2005;24(3):465–88. doi: 10.1016/j.jhealeco.2004.09.011 15811539 10.1016/j.jhealeco.2004.09.011

[22] Drummond MF, Sculpher MJ, Claxton K, Stoddart GL, Torrance GW. Methods for the Economic Evaluation of Health Care Programmes: Oxford University Press; 2005.

[23] Coto-Millán P, Inglada V. Essays on Transport Economics: Springer Science & Business Media; 2007.

[24] Beck-Friis B, Norberg H, Strang P. Cost analysis and ethical aspects of hospital-based home-care for terminal cancer patients. Scand J Prim Health Care. 1991;9(4):259–64. 1792451

[25] Board N, Brennan N, Caplan GA. A randomised controlled trial of the costs of hospital as compared with hospital in the home for acute medical patients. Aust N Z J Med. 2000;24(3):305–11. 10.1111/j.1467-842x.2000.tb01573.x 10937409

[26] Hughes S, Cummings J, Weaver F, Manheim L, Braun B, Conrad K. A randomized trial of the cost effectiveness of VA hospital-based home care for the terminally ill. Health Serv Res. 1992;26(6):801 1737710 PMC1069857

[27] Macintyre CR, Ruth D, Ansari Z. Hospital in the home is cost saving for appropriately selected patients: a comparison with in-hospital care. Int J Qual Health Care. 2002;14(4):285–93. 12201187 10.1093/intqhc/14.4.285

[28] Fan L, Lukin W, Zhao J, Sun J, Hou X-Y. Interventions targeting the elderly population to reduce emergency department utilisation: a literature review. Emerg Med J. 2015;32(9):738–43. doi: 10.1136/emermed-2014-203770 25527472 10.1136/emermed-2014-203770

[29] Saliba D, Kington R, Buchanan J, Bell R, Wang M, Lee M, et al Appropriateness of the decision to transfer nursing facility residents to the hospital. J Am Geriatr Soc. 2000;48(2):154–63. 10682944 10.1111/j.1532-5415.2000.tb03906.x

[30] Dranove D. An empirical study of a hospital-based home care program. Inquiry. 1985:59–66. 2998998

